# Reviewer acknowledgement 2012

**DOI:** 10.1186/1471-2105-14-80

**Published:** 2013-04-10

**Authors:** Catherine M Rice

**Affiliations:** 1 BioMed Central, 236 Gray’s Inn Road, London, WC1X 8HB, UK

## Abstract

**Contributing reviewers:**

The editors of BMC Bioinformatics would like to thank all our reviewers who have contributed to the journal in volume 13 (2012).

## 

Federico Abascal

Spain

Nuraini Abdul Rashid

Malaysia

Hidenao Abe

Japan

Vida Abedi

USA

Josep F Abril

Spain

Alexej Abyzov

USA

Jörg Ackermann

Germany

Antreas Afantitis

Cyprus

Estelle Afshar

USA

Pankaj Agarwal

USA

Shandar Ahmad

Japan

Joao Aires-De-Sousa

Portugal

Tero Aittokallio

Finland

Nikolaos Alachiotis

Germany

M.Mar Alba

Spain

Frank Alber

USA

Sonja-Verena Albers

Germany

Mario Albrecht

Germany

Raul Alcaraz Martinez

Spain

Alexander Alekseyenko

USA

Basim Alhadidi

Jordan

Can Alkan

USA

Eivind Almaas

Norway

Jonas Almeida

USA

Naomi Altman

USA

Kaushalya Amarasinghe

Australia

Fernando Amat

USA

Nancy Amato

USA

Li An

USA

Hyun Joo An

Korea, South

Sophia Ananiadou

UK

Nicola Ancona

Italy

Ebbe Sloth Andersen

Denmark

Bill Andreopoulos

Germany

Evelina Angov

USA

Richard Anney

Ireland

Maciek Antoniewicz

USA

Emilia Apostolova

USA

Sivaram Arabandi

USA

Kellie Archer

USA

Cecilia Arighi

USA

Masanori Arita

Japan

Jonathan Arthur

Australia

Kiyoshi Asai

Japan

Didier Auboeuf

France

Ludovic Autin

USA

Ferhat Ay

USA

Daniel Ayres

USA

M Madan Babu

UK

Michael Bada

USA

Andreu Badal

USA

Guy Baele

Belgium

Kristin Baetz

Canada

Vesselin Baev

Bulgaria

Ranjit Prasad Bahadur

India

Timothy Bailey

Australia

Christopher Baker

Canada

Rama Balakrishnan

USA

Pierre Baldi

USA

Sanghamitra Bandyopadhyay

India

Julio Banga

Spain

Pavel Baranov

Ireland

William Barbazuk

USA

Nuno L Barbosa-Morais

Canada

Pascal Barbry

France

J Christopher Bare

USA

Subhadip Basu

India

Timothy Bath

UK

Jan Baumbach

Germany

Scott Beatson

Australia

Mark Beaumont

UK

Tim Beck

UK

Tim Becker

Germany

Robert Beiko

Canada

Stephan Beisken

UK

Jennifer Below

USA

Henrik Bengtsson

USA

Tom Bennett

UK

Eric Bennett

USA

Igor Berezovsky

Norway

Fabrice Berger

Belgium

Frank Bergmann

USA

Juliana Bernardes

Brazil

Stephan Bernhart

Austria

Rameen Beroukhim

USA

Francois Berthiaume

USA

Miguel Betegon

USA

Gyan Bhanot

USA

Talapady Bhat

USA

Gaurav Bhatia

USA

Silvio Bicciato

Italy

Jacob Bien

USA

Jadwiga Bienkowska

USA

Joanna Biernacka

USA

Holly Bik

USA

Sylvain Billiard

France

Harald Binder

Germany

Hans Binder

Germany

Daniel Bindreither

Austria

Inanc Birol

Canada

Michael Black

New Zealand

Mathieu Blanchette

Canada

Marta Bleda

Spain

Christoph Bleidorn

Germany

Anna Block

USA

Jochen Blom

Germany

Christian Blouin

Canada

Xiaochen Bo

China

Alexander Bockmayr

Germany

Jens Boenigk

Germany

Jacky Bonaventure

France

Andrew Bordner

USA

Karsten Borgwardt

Germany

Marco Botta

Italy

Christina Boucher

USA

Jérôme Boulanger

France

Anne-Laure Boulesteix

Germany

Bastien Boussau

France

Arthur Brady

USA

Aaron Brandes

USA

Laurent Brehelin

France

Bronislava Brejova

Slovakia

Fiona Brinkman

Canada

Sylvain Brisse

France

Mitchell Brittnacher

USA

Luciano Brocchieri

USA

Mathias Brochhausen

USA

Guy Brock

USA

Jeremy Brown

USA

James Brown

USA

C. Titus Brown

USA

Sharon Browning

USA

Edgar Brunner

Germany

David Bryant

New Zealand

Michal Brylinski

USA

Mark Brynildsen

USA

Daniel Buchan

UK

Inbal Budowski-Tal

Israel

Robert Buels

USA

Nicolas Bruno Buisine

France

Janusz Bujnicki

Poland

Alexandre Bureau

Canada

Anthony Burgard

USA

Gully Burns

USA

Hauke Busch

Germany

Jutta Buschbom

Germany

Kimberly Bussey

USA

Chris Bystroff

USA

Mario Caccamo

UK

Gemma Cadby

Australia

Carmen Cadilla

USA

Yunpeng Cai

China

Yudong Cai

China

Raffaele Calogero

Italy

Laurence Calzone

France

Catarina Campbell

USA

Colin Campbell

USA

Nicola Cannata

Italy

Steven Cannon

USA

Zhiwei Cao

China

Minh Duc Cao

Australia

Natasha Caplen

USA

Michele Caraglia

Italy

Constanza Cardenas

Chile

Gabriel Cardona

Spain

Vincent Carey

USA

Joao Andre Carrico

Portugal

Kim Warwick Carter

Australia

Benilton Carvalho

UK

Rita Casadio

Italy

Robert Castelo

Spain

Frederic Cazals

France

Luigi Cerulo

Italy

Monica Chagoyen

Spain

Sutirtha Chakraborty

USA

Robert Chalkley

USA

Cliburn Chan

USA

Claudine Chaouiya

Portugal

Wendy Chapman

USA

Brad Chapman

USA

Charles Chapple

France

Andrew Chatr-Aryamontri

UK

Sidhartha Chaudhury

USA

Jennifer Chayes

USA

Liang Chen

USA

Ken Chen

USA

Xin Chen

Singapore

Brian Chen

USA

Haifeng Chen

China

Jianhan Chen

USA

Yu Zong Chen

Singapore

Chong-Jian Chen

France

Zhongxue Chen

USA

Yi-Hau Chen

Taiwan

Xiaowei Chen

USA

Mengjie Chen

USA

Jie Chen

USA

Luonan Chen

China

Xue-Wen Chen

USA

Chao Cheng

USA

Jianlin Cheng

USA

Cheng Cheng

USA

Feng Cheng

China

Yizong Cheng

USA

Lu Cheng

Finland

S Chakra Chennubhotla

USA

J Michael Cherry

USA

Kei-Hoi Cheung

USA

Derek Chiang

USA

Helene Chiapello

France

Marcus Chibucos

USA

Wai Ki Ching

Hong Kong

Fabien Chirot

France

Hamidreza Chitsaz

USA

Hyungwon Choi

Singapore

Nicolas Chopin

France

Bjorn E. Christensen

Norway

Hon Nian Chua

USA

Darya Chudova

USA

Elisa Cilia

Italy

Wyatt Clark

USA

Adrian F. Clark

UK

Vincent Claveau

France

Jose C. Clemente

USA

Timothy Clough

USA

Cristian Coarfa

USA

Daniel Coca

UK

Bradley Coe

USA

Luis Pedro Coelho

Portugal

Theresa Coetzer

South Africa

Avril Coghlan

Ireland

Kevin Cohen

USA

Ofir Cohen

Israel

Sarah Cohen-Boulakia

France

Lachlan Coin

UK

Jacques Colinge

Austria

Eric Collisson

USA

Christian Colmsee

Germany

Erin Conlon

USA

Karen Conneely

USA

Bruno Contreras-Moreira

Mexico

Leslie Cope

USA

Francesca Cordero

Italy

Eric Cosatto

USA

Richard G. Cote

UK

Laurent Cournac

France

Francisco Couto

Portugal

Lindsay Cowell

USA

Anthony Cox

UK

Maxime Crochemore

UK

Aedin Culhane

USA

Tobias Czauderna

Germany

Paulo Da Fonseca

Portugal

Alan Dabney

USA

Michal Dabrowski

Poland

Theodoros Damoulas

USA

Will Dampier

USA

Petr Danecek

UK

Tran Dang Hung

Japan

Ludmila Danilova

USA

Charles Danko

USA

Phuong Dao

Canada

Hatef Darabi

Sweden

Aaron Darling

USA

Rhiju Das

USA

Susmita Datta

USA

Sean Daugherty

USA

Xavier Daura

Spain

Lawrence David

USA

Sean Davis

USA

Noor Dawany

USA

Rajat De

India

Riccardo De Bin

Germany

Benjamin De Bivort

USA

Pieter De Bleser

Belgium

Alexandre De Brevern

France

Miquel De Cáceres Ainsa

Spain

Luis F De Figueiredo

UK

Xavier De La Cruz

Spain

Omar De La Cruz Cabrera

USA

Riet De Smet

Belgium

Anita De Waard

Netherlands

Charlotte Deane

UK

Daniela Delneri

UK

Francesca Demichelis

Italy

Zhigang Deng

USA

Youping Deng

USA

Minghua Deng

China

Ye Deng

USA

Narayan Desai

USA

Patrick Descombes

Switzerland

Alessandro Desideri

Italy

Christophe Dessimoz

Switzerland

Colin Dewey

USA

Raja Dey

USA

Christoph Dieterich

Germany

Antigone Dimas

Greece

Shuyan Ding

China

Irina Dinu

Canada

Gregory Ditzler

USA

Kevin Dobbin

USA

Alexander Dobin

USA

Jerry Dodgson

USA

Andrew Doig

UK

Ian John Donaldson

UK

Alexander Donath

Germany

Qunfeng Dong

USA

Philip Dormitzer

USA

Edward Dougherty

USA

Andrew Dowsey

UK

Finn Drabløs

Norway

Tom Druet

Belgium

Sumeet Dua

USA

Guangyou Duan

Germany

Christine Duarte

USA

Frank Dudbridge

UK

Stanislaw Dunin-Horkawicz

Poland

Adam Dupuy

USA

Julien Dutheil

France

Dent Earl

USA

Birgitta Elisabeth Ebert

Germany

Jeanette Eckel-Passow

USA

Sean Eddy

USA

Mikel Egana Aranguren

Spain

Michael Eiden

UK

Karen Eilbeck

USA

Birgit Eisenhaber

Singapore

Aron Eklund

Denmark

Yasser El-Manzalawy

USA

Laura Elo

Finland

Frank Emmert-Streib

UK

Burak Erman

Turkey

Adam Ertel

USA

Roger Estrada

Spain

Catherine Etchebest

France

Patricia Evans

Canada

Eduardo Eyras

Spain

Katti Faceli

Brazil

James Faeder

USA

Xiaodan Fan

Hong Kong

David Fardo

USA

Piero Fariselli

Italy

Joseph Fass

USA

Alexander Favorov

USA

Dmitry Fedorov

USA

K. Anton Feenstra

Netherlands

Jianxing Feng

China

Juan Fernandez-Recio

Spain

Fulvia Ferrazzi

Germany

Fabrizio Ferrè

Italy

Artur Ferreira

Portugal

Elana Fertig

USA

Ana Carolina Fierro

Belgium

Greg Finak

USA

Andras Fiser

USA

Patrick Flaherty

USA

Darren Flower

UK

Franklin Fondjo Fotou

USA

Charless Fowlkes

USA

David Fredman

Austria

Iddo Friedberg

USA

Dmitrij Frishman

Germany

Matt Fritz

USA

Holger Froehlich

Germany

Jon Froehlich

USA

Menachem Fromer

USA

Christian Fuchsberger

USA

Georg Fuellen

Germany

Andre Fujita

Japan

Laura Ines Furlong

Spain

Nicholas Furnham

UK

Chikara Furusawa

Japan

Monika Fuxreiter

Hungary

Ivan G. Costa

Brazil

Toni Gabaldon

Spain

Julien Gagneur

Germany

Mitchell Gail

USA

Giuliano Galimberti

Italy

Giuseppe Gallone

UK

Anna Gambin

Poland

Juntao Gao

China

Mu Gao

USA

Javier Garcia-Garcia

Spain

Vijay Garla

USA

Chad Garner

USA

Jean Garnier

France

Immacolata Garzilli

Italy

Olivier Gascuel

France

Christine Gaspin

France

Derek Gatherer

UK

Domenico Gatti

USA

Edward Gatzke

USA

Pascale Gaudet

USA

Huanying Ge

USA

Robin Genuer

France

Olivier Gevaert

USA

Viviana Giampaoli

Brazil

Michael Gilchrist

USA

Christian Gilissen

Netherlands

Walter Gilks

UK

Ryan Gill

USA

Jesse Gillis

Canada

Filip Ginter

Finland

Gonzalo Giribet

USA

Anthony Gitter

USA

Georgios Gkoutos

UK

Margaret Glasner

USA

Karl-Heinz Glatting

Germany

Peter Glaus

UK

Daniel Glez-Peña

Spain

Adam Godzik

USA

Jelle Goeman

Netherlands

Philip Goetz

USA

Derek Gordon

USA

Assaf Gordon

USA

Pawel Gorecki

Poland

Michael Gormley

USA

Susumu Goto

Japan

Osamu Gotoh

Japan

Mickael Goujon

UK

Manolo Gouy

France

Manfred Grabherr

USA

David Grant

USA

Gregory Grant

USA

Simon Gravel

USA

John Greally

USA

Casey Greene

USA

Sam Griffiths-Jones

UK

Michael Gromiha

India

Dominik Gront

Poland

Aleksandra Gruca

Poland

Stefan Grünewald

China

Jin Gu

China

Hong Gu

China

Shenheng Guan

USA

Weihua Guan

USA

Alain Guenoche

France

Rudy Guerra

USA

Vincent Guillemot

France

Stephane Guindon

New Zealand

Jun-Tao Guo

USA

Maozu Guo

China

Dinesh Gupta

India

Abhishekh Gupta

Finland

Metin Gurcan

USA

Attila Gursoy

Turkey

Daniel Gusenleitner

USA

Brian Haas

USA

Ryan Haasl

USA

Faraz Hach

Canada

Michael Hackenberg

Spain

Christine Hackett

UK

Amber Hackstadt

USA

Michiaki Hamada

Japan

Julia Handl

UK

Robert Handsaker

USA

Sridhar Hannenhalli

USA

Keith Hansen

USA

Jin-Kao Hao

France

Tong Hao

China

Thomas James Hardcastle

UK

Johanna Hardin

USA

Wolf-Dietrich Hardt

Switzerland

Solomon Harrar

USA

Elena Harris

USA

Andrew Harrison

UK

Sikander Hayat

Sweden

Yongqun He

USA

Xin He

USA

Jing He

USA

Xuming He

USA

Steffen Heber

USA

Dieter Heermann

Germany

Dominik Heider

Germany

Ines Heiland

Germany

Manuela Helmer-Citterich

Italy

Volkhard Helms

Germany

Christopher Henry

USA

James Hensman

UK

David Hernandez

Switzerland

Javier Herrero

UK

Ann Hess

USA

Robert Hettich

USA

Matthew Hibbs

USA

Paul Higgs

Canada

Jon Hill

UK

Blanca Himes

USA

Lynette Hirschman

USA

Jan Hiss

Switzerland

Thomas Hladish

USA

Shinn-Ying Ho

Taiwan

Yen-Yi Ho

USA

Lorin Hochstein

USA

Katharina Hoff

Germany

Nils Hoffmann

Germany

William Hogan

USA

Karsten Hokamp

Ireland

Michelle Holko

USA

Liisa Holm

Finland

Peter Holmans

UK

Changjin Hong

USA

Andreas Hoppe

Germany

Peter Hornbeck

USA

Roman Hornung

Germany

Steve Horvath

USA

Lin Hou

USA

Wen-Lian Hsu

Taiwan

Jianhua Hu

USA

Yueshan Hu

USA

Jianjun Hu

USA

Shen Hu

USA

Dawei Huang

USA

Bingding Huang

Germany

Heng Huang

USA

Xudong Huang

USA

Yao-Ting Huang

Taiwan

Yang Huang

USA

Jianhua Huang

USA

Sébastien Huet

France

Austin Hughes

USA

Jan Hummel

Germany

Jui-Hung Hung

USA

Sarah Hunter

UK

Hongwei Huo

China

Junguk Hur

USA

Susan Huse

USA

Van Anh Huynh-Thu

Belgium

Tae Hyun Hwang

USA

Howook Hwang

USA

Doug Hyatt

USA

Susan Idicula-Thomas

India

Iñaki Inza

Spain

Ilya Ioshikhes

Canada

Hemant Ishwaran

USA

Atiq Islam

USA

Rezarta Islamaj Dogan

USA

Masumi Itoh

Japan

Donald Jacobs

USA

Edwin Jacox

France

Samira Jaeger

Spain

Andrew Jaffe

USA

Samad Jahandideh

USA

Pooja Jain

UK

Tobias Jakobi

Germany

Hasan Jamil

USA

Woncheol Jang

Korea, South

Joël Janin

France

Vuk Janjic

UK

Jean-Luc Jannink

USA

Vivek Jayaswal

Australia

Hongkai Ji

USA

Zhenyu Jia

USA

Antonio José Yimeno Yepes

USA

Yuzhe Jin

USA

Xiangshu Jin

USA

Guangxu Jin

USA

Victor Jin

USA

Adam Johansen

UK

Peter Johansson

Australia

W. Evan Johnson

USA

Charles Johnson

USA

Inge Jonassen

Norway

Andrew Jones

UK

Clement Jonquet

France

Fredrik Jonsson

Sweden

Thomas Leonard Joseph

Singapore

Julie Josse

France

Fabrice Jossinet

France

Hsueh-Fen Juan

Taiwan

Richard Judson

USA

Chol-Hee Jung

Australia

Mayank Kabra

USA

Tim Kacprowski

Germany

Lars Kaderali

Germany

Andrey Kajava

France

Christoph Kaleta

Germany

Lukas Käll

Sweden

Kai Kammers

Germany

Zhongyuan Kan

USA

Michael Kane

USA

Seogchan Kang

USA

Dongwan Kang

USA

Kasthuri Kannan

USA

Natarajan Kannan

USA

Wei-Chun Kao

Taiwan

Emre Karakoc

USA

Peter Karp

USA

Yuliya Karpievitch

Austria

Masahiro Kasahara

Japan

Hisashi Kashima

Japan

Yuki Kato

Japan

Dukka Kc

USA

Birte Kehr

Germany

David Kelley

USA

Eamonn Keogh

USA

Andrew Kern

USA

Berit Kerner

USA

Daisuke Kihara

USA

Chang Sik Kim

UK

Sangtae Kim

USA

Jin-Dong Kim

Japan

Sun Kim

USA

Yohan Kim

USA

Jung-Jae Kim

Singapore

Seyoung Kim

USA

Choongrak Kim

Korea, South

Shuhei Kimura

Japan

Tobias Kind

USA

Carleton Kingsford

USA

Kengo Kinoshita

Japan

Martin Kircher

Germany

Jacob Kitzman

USA

Bernd Klaus

Germany

Judith Klein-Seetharaman

USA

Kjetil Klepper

Norway

Tomas Klingström

Sweden

David Klinke

USA

Andrzej Kloczkowski

USA

Rob Knight

USA

Uwe Koch

Germany

Ryotaro Koike

Japan

Felix Kokocinski

UK

Rachel Kolodny

Israel

Ravikumar Komandur Elayavilli

USA

Michal Komorowski

UK

Frank Konietschke

Germany

Rainer Konig

Germany

Kostas Konstantinidiss

USA

Dominik Kopczynski

Germany

Tamas Korcsmaros

Hungary

Sergey Koren

USA

Eduard Korkotian

Israel

Eija Korpelainen

Finland

Petros Kountouris

Cyprus

Mehmet Koyuturk

USA

Dima Kozakov

USA

Konstantinos Krampis

USA

Roland Krause

Germany

Arjun Krishnan

USA

Jens Olaf Krömer

Australia

Andriy Kryshtafovych

USA

Meghana Kshirsagar

USA

Rui Kuang

USA

Vipin Kumar

USA

Ursula Kummer

Germany

Sudip Kundu

India

Lukasz Kurgan

Canada

Maciej Kurpisz

Poland

Stefan Kurtz

Germany

Grzegorz Lach

Poland

Vincent Lacroix

France

Alain Laederach

USA

Xianyin Lai

USA

Yinglei Lai

USA

Philippe Laissue

UK

Tak-Wah Lam

Hong Kong

Sophie Lambert-Lacroix

France

Gabriel Landini

UK

Lydie Lane

Switzerland

Peter Langfelder

USA

Morgan Langille

USA

Benjamin Langmead

USA

Hilmar Lapp

USA

Roman Laskowski

UK

Richard Lathrop

USA

Charles Laughton

UK

Carolyn Lawrence

USA

Sébastien Lê

France

Kim-Anh Lê Cao

Australia

Robert Leaman

USA

Emilie Lebarbier

France

Richard Leduc

USA

Seungyeoun Lee

Korea, South

Chih Lee

USA

Yungling Lee

Taiwan

Sang Mee Lee

USA

Michael Lee

USA

Byungkook Lee

USA

Seunghak Lee

USA

Sunho Lee

Korea, South

Elliot Lefkowitz

USA

Heikki Lehvaslaiho

South Africa

Forian Leitner

Spain

Filip Leonarski

Poland

Paea Lependu

USA

Emmanuelle Lerat

France

Martin Lercher

Germany

Olivier Lespinet

France

Henry C.M. Leung

Hong Kong

Yaakov Levy

Israel

Hongzhe Li

USA

Sujun Li

China

Jun Li

USA

Weizhong Li

USA

Rui Li

China

Jinyan Li

Australia

Xia Li

China

Shuai Cheng Li

Canada

Feng Li

USA

Honglin Li

China

Xiaoli Li

Singapore

Bin Li

USA

Yun Li

USA

Kun Liang

USA

Alan Wee-Chung Liew

Australia

Markus Lill

USA

Michael Lin

USA

Nan Lin

USA

Heshan Lin

USA

Rongheng Lin

USA

Jake Lin

USA

Huiyi Lin

USA

Guohui Lin

Canada

Maurice Ling

Singapore

Thomas Lingner

Germany

Michal Linial

Israel

Simone Linz

Germany

Michael Liss

Germany

Tao Liu

USA

Haiyan Liu

China

Tzu-Yu Liu

USA

Xiaowen Liu

USA

Weiguo Liu

Singapore

Haibin Liu

USA

Bing Liu

Australia

Zhenqiu Liu

USA

Hui Liu

China

Jin Liu

USA

Hongfang Liu

USA

Peng Liu

USA

Jimin Liu

Singapore

Xiaole Liu

USA

Dennis Livesay

USA

Kevin Livingston

USA

Douglas Londono

USA

Tony Long

USA

Belen Lorente-Galdos

Spain

Doug Lorenz

USA

Riku Louhimo

Finland

Ed Louis

UK

Robert Lowe

UK

Todd Lowe

USA

Ari Loytynoja

UK

Yong Lu

USA

Chang-Tien Lu

USA

Zhiyong Lu

USA

Xinghua Lu

USA

Tu Luan

Norway

Dickson Lukose

Malaysia

Haiwei Luo

USA

Weijun Luo

USA

A Lynch

UK

David Lynn

Ireland

Eric Lyons

USA

Haisu Ma

USA

Shuangge Ma

USA

Ping Ma

USA

Li Ma

USA

Raghu Machiraju

USA

Richard Maclehose

USA

Tom Madden

USA

Matthew Maenner

USA

Stefan Renard Maetschke

Australia

Shaun Mahony

USA

Anup Mahurkar

USA

Izabela Makalowska

Poland

Vladimir Makarenkov

Canada

Vsevolod Makeev

Russian Federation

Takashi Makino

Japan

Yuko Makita

Japan

Anatoly Malevanets

Canada

James Malley

USA

Alberto Malovini

Italy

Brigitte Mangin

France

Ulrich Mansmann

Germany

Costas Maranas

USA

Antonio Marco

UK

Ignacio Marin

Spain

John Marioni

UK

Marianthi Markatou

USA

Florian Markowetz

UK

Pier Luigi Martelli

Italy

Vitor Martins Dos Santos

Netherlands

Marc A. Marti-Renom

Spain

Shinichiro Maruyama

Canada

Anna Maria Masci

USA

Andres R. Masegosa

Spain

Ali Masoudi-Nejad

Iran

Ewy Mathe

USA

Catherine Mathé

France

David Mathews

USA

Premendu P Mathur

India

Frederick Matsen

USA

Hirokazu Matsuda

Japan

Masafumi Matsuo

Japan

Manuel Mattheisen

USA

Melissa Matzke

USA

Ujjwal Maulik

India

Sebastian Maurer-Stroh

Singapore

Anoop Mayampurath

USA

Claus Mayer

UK

Daniel Mcduff

USA

Liam James Mcguffin

UK

Sean Mcilwain

USA

Bridget Mcinnes

USA

Brett Mckinney

USA

Geoff Mclachlan

Australia

Andrew Mcpherson

Canada

Paul Medvedev

Canada

Jens Meiler

USA

Peter Meinicke

Germany

Reinhard Meister

Germany

Jarek Meller

USA

Karyn Meltz Steinberg

USA

Guido Melzer

Germany

Renee X. De Menezes

Netherlands

Zhaoling Meng

USA

Bjoern Menze

USA

Enrique Merino

Mexico

Rainer Merkl

Germany

Wuttichai Mhuantong

Thailand

Tijana Milenkovic

USA

Jason Miller

USA

Webb Miller

USA

Ryan Mills

USA

Kazuharu Misawa

Japan

John Mitchell

UK

Sucharita Mitra

India

Makoto Miwa

UK

Qianxing Mo

USA

Beatrijs Moerkerke

Belgium

Pejman Mohammadi

Switzerland

Arumugam Mohana Priya

India

Mark Moll

USA

Stefano Monti

USA

Hojin Moon

USA

Sean Mooney

USA

Catherine Mooney

Ireland

Andrew D. Moore

Germany

Patrick S. Moore

USA

Roser Morante

Belgium

Fantine Mordelet

USA

Magali Moreau

USA

Martin Morgan

USA

Burkhard Morgenstern

Germany

Alexandre Morozov

USA

Giulia Morra

Italy

Melody Morris

USA

John Morris

USA

Jeffrey Morris

USA

Quaid Morris

Canada

David Morrison

Sweden

Giulia Morsica

Italy

Roberto Mosca

Germany

Harvey Motulsky

USA

Ross Mounce

UK

Jan Mrazek

USA

Matthieu Muffato

UK

Jennifer Mulle

USA

Wolfgang Müller

Germany

Conrad Mullineaux

UK

Christopher Mungall

USA

Adriana Munoz

Canada

Dragos Munteanu

USA

Chad Myers

USA

Niranjan Nagarajan

Singapore

Pradeep Naik

India

Shalima Nair

Australia

Achuthsankar Nair

India

Rafael Najmanovich

Canada

Kenta Nakai

Japan

Kensuke Nakamura

Japan

Masahiko Nakatsui

Japan

Sigve Nakken

Norway

Chanin Nantasenamat

Thailand

Arunachalam Narayanaswamy

USA

Tim W Nattkemper

Germany

Sven Nelander

Sweden

Martha Nelson

USA

Steffen Neumann

Germany

Pierre Neuvial

France

Bruno Nevado

Spain

Shu-Kay Ng

Australia

Cydney Nielsen

Canada

Mahesan Niranjan

UK

Hafumi Nishi

USA

Masha Niv

Israel

Jeffrey Noel

USA

Anja Nohe

USA

Houtan Noushmehr

USA

Maria Novatchkova

Austria

John Novembre

USA

Artem Novozhilov

USA

Gregory Nuel

France

Timothy Nugent

UK

Ruth Nussinov

USA

Brian Oakley

USA

Marcel Oberlaender

USA

Michael Ochs

USA

Anika Oellrich

UK

Bernard Offmann

France

Yanay Ofran

Israel

Heath Ogden

USA

Enno Ohlebusch

Germany

Yukinori Okada

USA

Michal Okoniewski

Switzerland

Constantin Orasan

UK

Christian Otto

Germany

Yu-Yen Ou

Taiwan

Anastasis Oulas

Greece

Zhengqing Ouyang

USA

Richard Owczarzy

USA

Lior Pachter

USA

Serguei Pakhomov

USA

Monica Palcic

Denmark

Xianming Pan

China

Anna Panchenko

USA

Gaurav Pandey

USA

Cheolwoo Park

USA

Taesung Park

Korea, South

Jong Park

Korea, South

Bogdan Pasaniuc

USA

Claude Pasquier

France

Jo-Ann Passmore

South Africa

Amrita Pati

USA

Ellis Patrick

Australia

Wayne Patrick

New Zealand

Georgios Pavlopoulos

Belgium

Marcin Pawlowski

USA

John Pearson

Australia

Shyamal Peddada

USA

Jimin Pei

USA

Eric Pelletier

France

Bo Peng

USA

Guy Perriere

France

Graziano Pesole

Italy

Joseph Petrosino

USA

Jonathan Pevsner

USA

Ernesto Picardi

Italy

Alexander Pico

USA

Lindenbaum Pierre

France

Vasyl Pihur

USA

Matias Piipari

UK

Armando Pinho

Portugal

Daniel Pinkel

USA

John W Pinney

UK

Roger Pique-Regi

USA

Walter Pirovano

Netherlands

Esa Pitkänen

Finland

Jason Pitt

USA

Rimma Pivovarov

USA

Matthias Platzer

Germany

Michael Poidinger

Singapore

Panayiota Poirazi

Greece

Thomas Pommier

France

Mihai Pop

USA

Alexey Porollo

USA

Christine Porzelius

Germany

Stephan Preibisch

USA

Robert Preissner

Germany

Nathan Price

USA

Cristin Print

New Zealand

Sebastian Proost

Belgium

Igor Pruenster

Italy

Kim Pruitt

USA

Teresa Przytycka

USA

Pere Puigbo

USA

Sampo Pyysalo

Japan

Jiang Qian

USA

Wei-Jun Qian

USA

Sanbo Qin

USA

Peng Qiu

USA

Weiliang Qiu

USA

Hong Qu

China

Liang-Hu Qu

China

Enrique Querol

Spain

Aaron Quinlan

USA

Richard Röttger

Germany

Predrag Radivojac

USA

Bernhard Radlwimmer

Germany

Gajendra Raghava

India

Emanuele Raineri

Spain

Satwik Rajaram

USA

Sanguthevar Rajasekaran

USA

Stuart Alexander Ralph

Australia

Matteo Ramazzotti

Italy

Milan Randic

USA

Juan A.G. Ranea

Spain

Huzefa Rangwala

USA

Mattias Rantalainen

UK

Shaoqi Rao

China

Arvind Rao

USA

Franck Rapaport

USA

Naim Rashid

USA

Suren Rathnayake

Australia

Oliver Ratmann

UK

Tobias Rausch

Germany

Simon Rayner

China

Dietrich Rebholz-Schuhmann

Switzerland

Pedro Reche

Spain

Martin Reczko

Greece

Juri Reimand

Canada

Mark Reimers

USA

Maido Remm

Estonia

Bernhard Renard

Germany

Osbaldo Resendis-Antonio

Mexico

Daniel Restrepo

Colombia

Adam Retchless

USA

Oleg Reva

South Africa

Alejandro Reyes

Germany

Christopher Reynolds

UK

Brooke Rhead

USA

Daniel Rigden

UK

Pentti Tapio Riikonen

Finland

Fabio Rinaldi

Switzerland

Daniel Ripoll

USA

Matthew Ritchie

Australia

Alberto Riva

USA

Guillaume Rizk

France

Stephane Robin

France

Mark Robinson

Switzerland

Peter Robinson

Germany

Marc Robinson-Rechavi

Switzerland

Philippe Rocca-Serra

UK

Nicolas Rodrigue

Canada

Maira Rodrigues

Brazil

Simon Rogers

UK

Torbjorn Rognes

Norway

F.James Rohlf

USA

Legier Rojas

Puerto Rico

Koerner Roman

Georgia

Elvira Romera

Spain

Peter Rose

USA

Gail Rosen

USA

Volker Roth

Switzerland

Kistian Rother

Germany

Eric Rouchka

USA

Jacques Rougemont

Switzerland

Marc Roussel

Canada

Juho Rousu

Finland

Maga Rowicka

USA

Ruth Rowland

UK

Witold Rudnicki

Poland

Oscar M Rueda

UK

Anna Rumshisky

USA

David Russell

USA

Mirabela Rusu

USA

Christopher Ryu

USA

Andrey Rzhetsky

USA

Smitha S S

India

Chiara Sabatti

USA

Gustavo Sacomoto

France

Pål Sætrom

Norway

Yvan Saeys

Belgium

Carleton Sage

USA

Sudipto Saha

India

Satya Sahoo

USA

Hiroto Saigo

Japan

Gabriele Sales

Italy

Andrej Sali

USA

Leena Salmela

Finland

Manuel Salvadores

USA

Steven Salzberg

USA

Adeline Samson

France

Matthias Samwald

Austria

Jeffry Sander

USA

Kuljeet Sandhu

India

Roman Sandler

Israel

Rodrigo Santamaría

Spain

Monica Santamaria

Italy

Guillaume Santini

France

Mihaela Sardiu

USA

Ugis Sarkans

UK

Indra Neil Sarkar

USA

Anindya Sarkar

USA

Maureen Sartor

USA

Kengo Sato

Japan

Tetsuya Sato

Japan

Richard Savage

UK

Eric Sayers

USA

Alejandro Schaffer

USA

Leonard Schalkwyk

UK

Theresa Scharl

Austria

Oliver Schilling

Germany

Markus Schirle

USA

Alexander Schleiffer

Austria

Andreas Schlicker

Netherlands

Patrick Schloss

Germany

Matthias Schmid

Germany

Reinhard Schneider

Luxembourg

Dina Schneidman

USA

Alexander Schoenhuth

Netherlands

Paul Schofield

UK

Christian Schönbach

Japan

Fabian Schreiber

UK

Michael Schroeder

Germany

Martijn Schuemie

Netherlands

Stefan Schulz

Germany

Marcel H. Schulz

USA

Jean-Marc Schwartz

UK

Roland Schwarz

UK

Benno Schwikowski

France

Celine Scornavacca

France

Harvey Morgan Scott

USA

Ridgway Scott

USA

Marco Scutari

UK

Alexander Sczyrba

Germany

Stefan Seemann

Germany

Michael Seifert

Germany

Joachim Selbig

Germany

Junhee Seok

USA

Oliver Serang

USA

Jun Sese

Japan

Joao Setubal

Brazil

Babak Shahbaba

USA

Jahangheer Shaik

USA

Hugh Shanahan

UK

Maxim Shatsky

USA

Shula Shazman

USA

Ekaterina Shelest

Germany

Yaoqing Shen

Canada

Bairong Shen

China

Yufeng Shen

USA

Hong-Bin Shen

China

Jamie Sherman

Australia

Shuoyong Shi

USA

Wei Shi

Australia

Tetsuo Shibuya

Japan

Yin Shugart

USA

Maulik Shukla

USA

Mark Edward Siddall

USA

John Sidney

USA

Kimberly Siegmund

USA

Fabian Sievers

Ireland

Mikko J. Sillanpaa

Finland

Noah Simon

USA

Thomas Simonson

France

Jared Simpson

UK

Suzanne Sindi

USA

Mona Singh

USA

Nicholas Sinnott-Armstrong

USA

Jeffrey Skolnick

USA

Marcin J. Skwark

Sweden

Montgomery Slatkin

USA

Kevin Small

USA

Barry Smith

USA

Marjolein Smith

USA

Gordon Smyth

Australia

Christopher Snow

USA

Dinesh Soares

UK

Johannes Soding

Germany

Sunghwan Sohn

USA

Stian Soiland-Reyes

UK

Henry Soldano

France

Hamid Soltanian-Zadeh

USA

Imre Solti

USA

Charlotte Soneson

Switzerland

Jiangning Song

Australia

Chi Song

USA

Silke Sperling

Germany

Andrej-Nikolai Spiess

Germany

Marina Spivak

USA

Ola Spjuth

Sweden

Andrea Splendiani

UK

John L Spouge

USA

Padmini Srinivasan

USA

Johan Staaf

Sweden

Alexandros Stamatakis

Germany

Dan Stanzione

USA

Alexander Statnikov

USA

Mike Steel

New Zealand

Gerhard Steger

Germany

Oliver Stegle

Germany

Kathleen Steinhofel

UK

Dov Joseph Stekel

UK

Ulrich Stelzl

Germany

Michael Stewart

UK

Vilberto Stocchi

Italy

Aleksandar Stojmirovic

USA

Margaret-Anne Storey

Canada

Bruno Studer

Denmark

Zhengchang Su

USA

Isaac Subirana

Spain

Marc Suchard

USA

Peter Sudmant

USA

Frank Suits

USA

Pavel Sumazin

USA

Ron Summers

UK

Yijun Sun

USA

Edward Susko

Canada

Shingo Suzuki

Japan

Neil Swainston

UK

Jason Swedlow

UK

Krister Swenson

Canada

Zoltan Szallasi

USA

Sing-Hoi Sze

USA

Radek Szklarczyk

Netherlands

David Tabb

USA

Y-H. Taguchi

Japan

Leila Taher

Germany

Takeyuki Tamura

Japan

Kailin Tang

China

Haibao Tang

USA

Navdeep Tangri

Canada

Eric Tannier

France

Ralf Tautenhahn

USA

James Taylor

USA

Ronald Taylor

USA

Marion Dawn Teare

UK

Vladimir Teif

Germany

Waibhav Tembe

USA

Douglas Teodoro

Switzerland

Thomas Terwilliger

USA

Bas Teusink

Netherlands

Fabian J Theis

Germany

Kristof Theys

Belgium

Paul Thomas

USA

Stephen Thomas

France

Roman Thomas

Germany

Julie Thompson

France

Julie Dawn Thompson

France

David Thybert

UK

Bruce Tidor

USA

Elisabeth Tillier

Canada

Jean-Yves Tinevez

France

Hannah Tipney

UK

Toshiaki Tokimatsu

Japan

Michael Tolstorukov

USA

Yigang Tong

China

Manabu Torii

USA

Silvio Tosatto

Italy

Nora Toussaint

USA

Christopher Town

UK

Zlatko Trajanoski

Austria

Anna Tramontano

Italy

Leon-Charles Tranchevent

Belgium

William Trimble

USA

Carl Troein

Sweden

Jerry Tsai

USA

Richard Tzong-Han Tsai

Taiwan

George Tsatsaronis

Germany

Alex Tsodikov

USA

Allan Tucker

UK

Chun-Wei Tung

Taiwan

Ernest Turro

UK

Michael Tyers

Canada

Ikuo Uchiyama

Japan

Graham Upton

UK

Filippo Utro

USA

Vladimir Uversky

USA

Sandor Vajda

USA

Niko Välimäki

Finland

Giorgio Valle

Italy

Harm Van Bakel

Canada

Michiel Van Bel

Belgium

Alena Van Bömmel

Germany

Mark Van De Wiel

Netherlands

Katrijn Van Deun

Belgium

Gary Van Domselaar

Canada

Stijn Van Dongen

UK

Leo Van Iersel

Netherlands

Maarten Van Iterson

Netherlands

Sofie Van Landeghem

Belgium

Peter Van Loo

Belgium

Erik Van Mulligen

Netherlands

Vincent Vanburen

USA

Klaas Vandepoele

Belgium

Fabio Vandin

USA

Alexei Vazquez

USA

Krishna Veeramah

USA

Ricardo Vencio

Brazil

Fons Verbeek

Netherlands

Stella Veretnik

USA

Nicolas Vergne

France

Vanessa Vermeirssen

Belgium

George Vernikos

Greece

Karin Verspoor

USA

Christoph Viebahn

Germany

Jorge Vila

USA

Nicola Vitulo

Italy

Juan Antonio Vizcaino

UK

Ioannis Vlachos

Greece

Arndt Von Haeseler

Austria

Slobodan Vucetic

USA

Stephan Waack

Germany

Thomas Waechter

Germany

Jerome Waldispuhl

Canada

Levi Waldron

USA

Alan Walker

UK

Iain Wallace

USA

Bjorn Wallner

Sweden

Ian Walsh

Italy

Dirk Walther

Germany

Xiang Wan

Hong Kong

Yinan Wan

China

Lin Wan

USA

Xiu-Feng (Henry) Wan

USA

Kai Wang

USA

Rui-Sheng Wang

USA

Xiaofeng Wang

USA

Xiaoming Wang

Canada

Difei Wang

USA

Penghao Wang

Australia

Guangshun Wang

USA

Haiying Wang

UK

Wei Wang

USA

Chaolong Wang

USA

Ting Wang

China

Renxiao Wang

China

Xiaowo Wang

China

Haiyan Wang

USA

Hongning Wang

China

Xiaosheng Wang

USA

Dierk Wanke

Germany

Michael Washburn

USA

Nicole Washington

USA

Mark Wass

Spain

Josh Waterfall

USA

Bruce Watson

South Africa

Bonnie Webber

UK

Lawrence Wee

Singapore

Louis Wehenkel

Belgium

Zhi Wei

USA

Kaifa Wei

China

Chaochun Wei

China

Martin Weigt

France

Danielle Welter

UK

Lorenz Wernisch

UK

Lodewyk Wessels

Netherlands

Sean West

USA

Johan Westerhuis

Netherlands

Oscar Westesson

USA

Paul Whitford

USA

Susann Wicke

Germany

Bartek Wilczynski

Poland

Mark Wilkinson

UK

Sebastian Will

USA

Rob Willems

Netherlands

Rohan Williams

Singapore

Stacey Winham

USA

Dave Winkler

Australia

Martyn Winn

UK

Rainer Winnenburg

USA

Ole Winther

Denmark

Elizabeth Winzeler

USA

Tobias Wittkop

USA

Svante Wold

USA

Yuri Wolf

USA

Olaf Wolkenhauer

Germany

Sungho Won

Korea, South

Limsoon Wong

Singapore

Sangsoon Woo

USA

Jonathan Wren

USA

Steven Wu

USA

Yufeng Wu

USA

Hao Wu

USA

Zhijin Wu

USA

Stephen Wu

USA

Fang-Xiang Wu

Canada

Michael Wu

USA

Zheyang Wu

USA

Qiangwei Xia

USA

Li Xia

USA

Qian Xiang

China

Guanghua Xiao

USA

Yuanyuan Xiao

USA

Xuan Xiao

China

Xiang-Qun Xie

USA

Lu Xie

China

Min Xu

USA

Guoqiang Xu

USA

Jinbo Xu

USA

Dong Xu

USA

Jun Xu

China

Qikai Xu

USA

Chenghai Xue

China

Nianwen Xue

USA

Richard Yamada

USA

Takashi Yamamoto

Japan

Yoshihiro Yamanishi

Japan

Xiting Yan

USA

Shao-Nian Yang

Sweden

Yuedong Yang

USA

Can Yang

USA

Wanling Yang

Hong Kong

Hsin-Chou Yang

Taiwan

Jinn-Moon Yang

Taiwan

Chen Yanover

USA

Xiaojun Yao

China

Christopher Yau

UK

Gokhan Yavas

USA

Omer Nebil Yaveroglu

UK

Yuzhen Ye

USA

Jian Ye

USA

Lana Yeganova

USA

Illhoi Yoo

USA

Joo-Yeon Yoo

Korea, South

Seungtai Yoon

USA

Ken Youens-Clark

USA

Laurent Younes

USA

Tao Yu

Singapore

Weichuan Yu

Hong Kong

Chenggang Yu

USA

Xueping Yu

USA

Yang Yu

USA

Kai Yu

USA

Feng Yue

USA

Eleni Zacharia

Greece

René Zahedi

Germany

Lurdes Zamora

Spain

Chongzhi Zang

USA

Hossein Zare

USA

Riccardo Zecchina

Italy

Daniel Zerbino

UK

Xianquan Zhan

China

Peng Zhang

USA

Bing Zhang

USA

Songmao Zhang

China

Yuping Zhang

USA

Bai Zhang

USA

Ziding Zhang

China

Louxin Zhang

Singapore

Haibin Zhang

USA

Xian Zhang

USA

Tuo Zhang

USA

Xiangde Zhang

China

Jinfeng Zhang

USA

Kui Zhang

USA

Hong Zhang

China

Hua Zhang

China

Zhongqi Zhang

USA

Yong Zhang

China

Jingfen Zhang

USA

Dabao Zhang

USA

Hao Zhao

USA

Xingming Zhao

China

Xiuwen Zheng

USA

Xiaoqi Zheng

China

Jim Zheng

USA

Chunhou Zheng

China

Degui Zhi

USA

Xiaobo Zhou

USA

Qing Zhou

USA

Huanyu Zhou

USA

Jie Zhou

USA

Yaoqi Zhou

USA

Lihua Zhu

USA

Bin Zhu

USA

Jiang Zhu

USA

Arthur Zimek

Germany

Ralf Zimmer

Germany

Jaroslaw Zola

USA

Xiaoqin Zou

USA

Pierre Zweigenbaum

France

